# Kinetic modeling of the sesamin conversion into asarinin in the presence of citric acid loading on Hβ

**DOI:** 10.3389/fnut.2022.983843

**Published:** 2022-08-10

**Authors:** Qiong Yu, Xiao-shuang Cai, Sébastien Leveneur, Xue-de Wang, Hua-min Liu, Chen-xia Zhang, Yu-xiang Ma

**Affiliations:** ^1^College of Food Science and Engineering & Institute of Special Oilseed Processing and Technology, Henan University of Technology, Zhengzhou, China; ^2^INSA Rouen, UNIROUEN, LSPC, UR4704, Normandie Université, Rouen, France

**Keywords:** sesamin, asarinin, catalyst, sesame oil, kinetic modeling

## Abstract

In the present work, effects of reaction temperature, reactant concentration, catalyst loading, and rotation speed on the kinetics of sesamin conversion in a sesame oil system were studied by using citric acid loading on Hβ zeolite (CA/Hβ) as a catalyst. A kinetic model was built for sesamin conversion. The kinetic model fits correctly the experimental concentration of sesamin and asarinin ($R_{S\,e\,s\,a\,m\,i\,n}^{2}$ = 0.93 and $R_{A\,s\,a\,r\,i\,n\,i\,n}^{2}$ = 0.97). The sesamin conversion is an endothermic reaction (△*H*_*r*Iso_ = 3 4.578*kJ*/*mol*). The CA/Hβ catalyst could be easily regenerated by calcination, and there was no obvious loss of catalytic activity when reused. Knowledge of the sesamin conversion is of great significance for guiding production and improving the value and nutrition of sesame oil. In a word, this study lays the foundation for the scale-up of the production of asarinin from sesame oil using CA/Hβ as the catalyst.

## Introduction

Lignans have been shown to possess a variety of physiological activities (1–3). Asarinin is a furofuran lignan. According to reports, compared to sesamin, asarinin has strong physiological activities such as anti-cancer, anti-proliferative (4), antioxidative (5), and antibacterial. It was reported that asarinin was yielded when sesamin was oxidized by potassium permanganate under acidic conditions. It was found that at the same concentration, the inhibition rate of asarinin on DPPH free radical reached 43.3%, while the inhibition rate of sesamin is 27.2% (5). Kim et al. (6) reported that asarinin and sesamin showed cytotoxicity with IC_50_ values of 67.25 and 98.57 μM, respectively, in MCF-7 cells. It was found that asarinin can increase the activity and gene expression of fatty acid oxidation enzymes of rats more strongly than sesamin (3). Asarinin has the potential to be clinically applied to induce cancer cell death and inhibit metastasis because of its antiangiogenic properties (7).

Asarinin is a geometrical isomer of sesamin generated under certain conditions of temperature and acidity (8). It is formed from sesamin during refining processes (i.e., acid clay bleaching and deodorization at high temperature) (9, 10). Compared with homogeneous catalysts (e.g., hydrochloric acid and sulfuric acid), heterogeneous catalysts such as phosphotungstic acid (11), acid cation exchange resin (12), acid clay (13), and Hβ zeolite (14) have the advantages of easier separation and recycling. Owing to its surface acidity and special porous structure, zeolite beta is a commonly used solid acid catalyst (SACs). Zeolite beta is widely applied in refining and in the chemical industry for various reactions such as isomerization, esterification, and alkylation (15–17). The maximum conversion rates for esterification of oleic acid and transesterification of soybean oil by the use of modified Hβ zeolite were 86% and 95%, respectively. The catalyst can be recycled up to four times without any loss during the conversion process (18).

**FIGURE 1:**
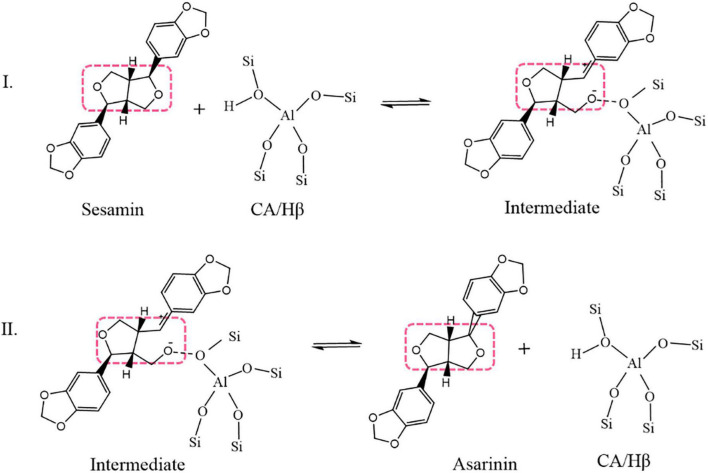
Simplified mechanism of sesamin conversion in sesame oil (dotted boxes enclose tetrahydrofuran rings).

The mechanism of sesamin conversion in a sesame oil system is illustrated in Figure 1 (8, 10). In a sesame oil system, CA/Hβ attacks the O atom on the tetrahydrofuran ring of sesamin and absorbs it into the active center of CA/Hβ. An unstable intermediate transition state forms in this process. Asarinin is produced through the desorption of the intermediate transition state; at the same time, CA/Hβ is regenerated.

For practical application, it is vital to be able to evaluate and predict the changes of sesame lignans under process conditions. Consequently, a reliable kinetic model is often needed. While there have been studies of the kinetics of the lipid peroxidation (19, 20) of vegetable oils and of biodiesel fuel (21) production from vegetable oils, the kinetics of sesamin conversion in a sesame oil system have not been studies. One research group noticed that the kinetics of sesamin conversion is of first-order in the ethanol-sesamin system by using hydrochloric acid as a catalyst (22). To the best of our knowledge, no kinetic models have been developed for the sesamin conversion in sesame oil system. The SACs studied here, which was used in our previous work, showed good catalytic performance (14, 23). In this study, the kinetics of the sesamin conversion with CA/Hβ in cold-pressed sesame oil (CSO) was investigated. Influences of reaction parameters including temperature, reactant concentration, catalyst loading, and rotation speed on the kinetics of sesamin conversion were evaluated. Kinetic modeling was developed to estimate the rate constants and activation energy of sesamin conversion with the catalyst CA/Hβ under these reaction conditions. This study provides a theoretical foundation for controlling conditions promoting the sesamin conversion, and the mass production of high-quality sesame oils rich in asarinin.

## Experimental section

### Materials and chemicals

Chromatographic-grade methanol was obtained from VBS Biologic INC, United States. Hydrogen type of zeolite beta with a Si/Al ratio of 25 (Hβ) was purchased from the Nankai University catalyst plant, Tianjin, China. Sesamin was obtained from Macklin Biochemical Co., Ltd., Shanghai, China. Asarinin was purchased from Purechem-standard Co., Ltd., Chengdu, China. Citric acid (CA) was obtained from Shanghai Yuanye Biotechnology Co., Ltd., Shanghai, China. The standard substances were stored at 4°C in darkness. CSO was made in a hydraulic press (Bafang Ltd., model XL-600, Suzhou, China) in our laboratory.

### Preparation of sesame oil samples

Considering the content and the solubility of sesamin in sesame oil, a certain amount of sesamin (30 and 60 mg) was added to 70 g sesame oil, and then the mixture was treated with ultrasound for 30 min, subsequently, the mixture was placed in a thermostatic magnetic water bath stirred for 4 h at 37°C (24). Sesamin and asarinin contents in all sesame oil samples were analyzed using HPLC-UV (high-performance liquid chromatography combined with ultraviolet detector) methods based on the pervious literature (25, 26). The sesamin conversion (%) was calculated using the following equation:


$$\text{sesamin}\,\text{conversion}\%=\left(1-\frac{{\text{C}}_{A}}{{\text{C}}_{\mathrm{A0}}}\right)\times 100$$

where, C_*A0*_ (mg/100 g) is the initial concentration of sesamin in the sesame oil, and C_*A*_ (mg/100 g) is the sesamin concentration at a particular time in the reaction process.

### Preparation of catalyst

Citric acid loading on Hβ was prepared on the basis of the reported literature (17, 23, 27). Briefly, a 20 g sample of Hβ was added to 200 ml of critic acid aqueous solution, and the resulting suspension was stirred at room temperature for 16 h. And then washed, dried and calcined. The catalyst has been proven to promote the conversion of sesamin into asarinin and shows well activity (14).

### Apparatus and experimental procedures

**FIGURE 2:**
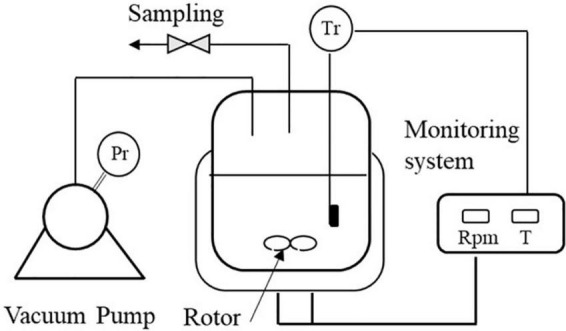
Schematic of reaction setup.

The reaction apparatus is shown in Figure 2. Experiments were performed in a 250 ml glass-jacket reactor equipped with a magnetic stirrer, vacuum pump and temperature control system.

### Kinetic study of sesamin conversion

In total, 50 g of CSO was added to the 250 ml glass-jacket reactor at a certain stirring speed (see Table 1). When the oil temperature reached the set temperature, a specific amount of catalyst was added into the reactor vessel. Agitation was started. 2.0 ml sesame oil samples were collected at specific time points. The sesame oil sample was immediately placed in an ice water bath to quench the reaction and then centrifuged. The sesame oil samples were stored at −20°C for analysis within 2 days.

**TABLE 1:** Experimental matrix for the sesamin conversion.

Run	Initial reactant C_*A*_	*T*	Catalyst loading	Rotation speed
	mmol/L	°C	wt.%	rpm
1	21.47	80	1.6	300
2	23.83	80	1.6	300
3	22.91	80	1.6	300
4	21.47	70	1.6	300
5	21.47	90	1.6	300
6	21.47	80	1.0	300
7	21.47	80	3.0	300
8	21.47	80	1.6	200
9	21.47	80	1.6	400

Table 1 displays the experimental matrix for each run with the detailed reaction conditions. The parameters affecting sesamin conversion of sesame oil were reaction temperature (70–90°C), initial concentration of sesamin (21.47–23.83 mmol/L), catalyst loading (1.0–3.0%) and rotation speed (200–400 rpm). These parameters were selected according to the results of previous tests (14). C_*A*_ (mg/100 g) and C_*B*_ (mg/100 g) represent the concentrations of sesamin and asarinin, respectively, in the oil samples during the whole reaction process. At the beginning of all kinetic experiments, there was no asarinin; consequently, the concentration of asarinin was zero at time zero.

### Recycling of the catalyst

After execution of the conversion reaction, the reaction mixture was centrifuged at 4,500 rpm for 20 min. The CA/Hβ catalyst was separated, washed with n-hexane and dried in an oven at 60°C, and then, the recycled catalyst was calcined in a muffle furnace (500°C) for 4 h. The reused catalyst was characterized by N_2_ adsorption and desorption isotherm analysis (Micromeritics, 3-Flex, United States). The surface areas of catalysts were calculated by BET method using adsorption data (28).

### Modeling

Athena Visual Studio software was used for parameter estimation (29, 30). During the kinetic modeling stage, the concentrations of sesamin and asarinin were used as observables. DDAPLUS solver, included in Athena Visual Studio, integrates ordinary differential equations (ODEs) (10, 11). This solver is a modified Newton algorithm used in conjunction with a fixed leading coefficient backward difference formula for the approximation of first-order derivatives (31). In this study, the modeling dealt with a multi-response parameter estimation. The GREGPLUS subroutine package included in Athena Visual Studio was applied to minimize the objective function *S*(θ), and calculate the maximum posterior probability density of the various estimated parameters and the values of the posterior distribution of the tested models (30). The objective function was defined as


(1) S⁢(θ)=(n+m+1)⋅l⁢n⁢|υ⁢(θ)|

where, *m* is the number of responses, *n* is the number of events in response and |υ(θ)| is the determinant of the covariance matrix of the responses. Each element of this matrix is denoted as:


(2) $$\upsilon_{i\,j}\,\left(\theta \right)=\sum_{u=1}^{n}\left[Y_{i\,u}-Y_{i\,u}\,\left(\theta \right)\right]\cdot \left[Y_{j\,u}-Y_{j\,u}\,\left(\theta \right)\right]$$

with *Y*_*iu*_ the experimental concentration and *Y*_*iu*_(θ) the estimated value for the response *i*, and event *u*; *Y*_*ju*_is the experimental concentration, *via* the estimated parameters θ, *Y*_*ju*_(θ) is the estimated value for the response *j*, and event *u*. The precision of the estimated parameters was evaluated by the 95% marginal highest posterior density (HPD). The 95% HPD was calculated by the GREGPLUS package.

### Statistical analysis

All measurements were carried out in triplicate and the data were expressed as mean values.

## Results and discussion

### Kinetic study

**FIGURE 3:**
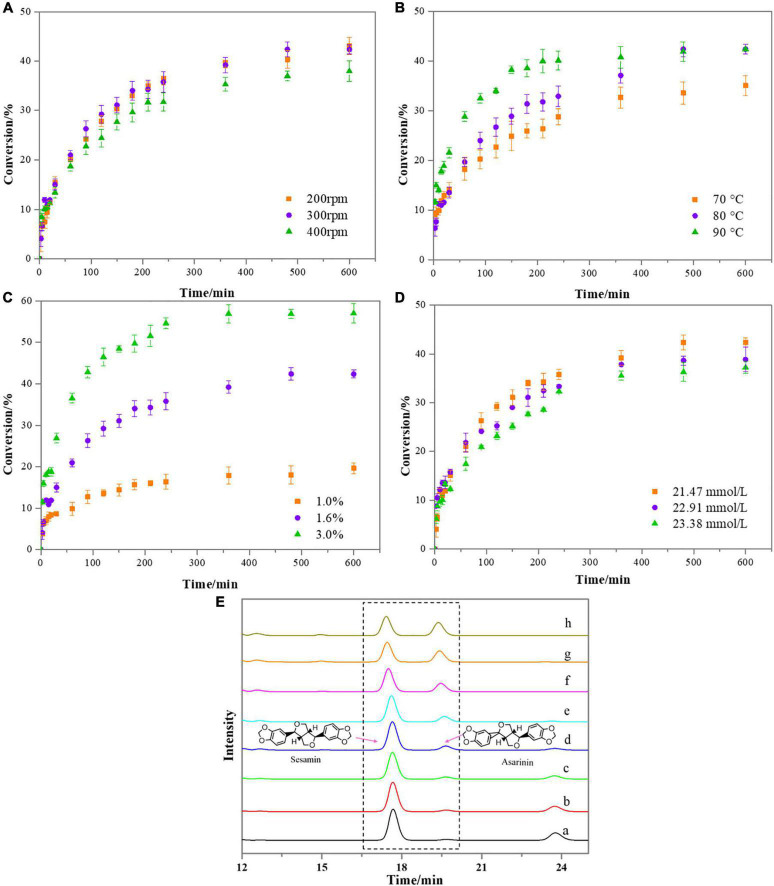
Effect of reaction conditions on the kinetics of the sesamin conversion under the following conditions: **(A)** catalyst loading, 1.6%; sesame oil temperature, 80°C; reactant concentration, 21.47 mmol/L; **(B)** catalyst loading, 1.6%; rotation speed, 300 rpm; reactant concentration, 21.47 mmol/L; **(C)** sesame oil temperature, 80°C; rotation speed, 300 rpm; reactant concentration, 21.47 mmol/L. **(D)** Catalyst loading, 1.6%; sesame oil temperature, 80°C; rotation speed, 300 rpm. Bars represent standard deviation (*n* = 3). **(E)** Variation in absorbance intensity of sesamin and asarinin in sesame oil samples at different reaction times under the following conditions: catalyst loading, 3.0%; sesame oil temperature, 80°C; rotation speed, 300 rpm; reactant concentration, 21.47 mmol/L (a, 3 min; b, 5 min; c, 10 min; d, 20 min; e, 30 min; f, 1.0 h; g, 2.0 h; h, 3.0 h).

In this section, the effect of reaction temperature (70–90°C), catalyst loading (1.0–3.0%), rotation speed (200–400 rpm) and initial concentrations of reactant (21.47, 22.91, and 23.38 mmol/L) on the kinetics of sesamin conversion were investigated. The sesamin conversion was selected as a response value, and its kinetic curves are plotted in Figures 3A–D.

### Effect of rotation speed

Three levels of rotation speeds (200, 300, and 400 rpm) were applied to reveal the influence of external mass transfer on the reaction kinetics. Experiments were performed under the following conditions: sesame oil temperature, 80°C, catalyst (CA/Hβ) loading, 1.6% and initial concentration of reactant, 21.47 mmol/L (Figure 3A). As the reaction proceed, the conversion of sesamin became lower. Experiments conducted at 200, 300, or 400 rpm displayed similar kinetics. There was no significant difference in sesamin conversion at 200 and 300 rpm. The conversion of sesamin decreased slightly when the rotation speed was 400 rpm. The stirring speed was found to have minor effects on the kinetics of sesamin conversion. The reason might be related to contact between the reactants and the active surface of the catalyst. This phenomenon is similar to the results reported previously (32).

### Effect of reaction temperature

The effect of reaction temperature (70, 80, and 90°C) on the sesamin conversion in sesame oil was investigated. The reaction parameters were as follows: catalyst (CA/Hβ) loading, 1.6%; rotation speed, 300 rpm; initial concentration of reactant, 21.47 mmol/L. As illustrated in Figure 3B, as the reaction temperature increased from 70 to 90°C, the conversion rate of sesamin also significantly increased. This phenomenon has been observed in previous studies when inorganic acid was used as catalyst for the sesamin conversion in an ethanol system (22). In general, high temperature reduces the viscosity of the oil and thus increases the mass transfer rate (33, 34). At the reaction time of 240 min, the sesamin conversion increased with temperature, as follows: 23.72% (70°C) < 35.78% (80°C) < 40.09% (90°C).

### Effect of catalyst loading

The influence of catalyst loading on sesamin conversion in sesame oil samples is illustrated in Figure 3C. The catalyst loading at three levels (1.0, 1.6, and 3.0% of sesame oil) were studied, using the following reaction conditions: sesame oil temperature, 80°C; initial concentration of reactant, 21.47 mmol/L; rotation speed, 300 rpm. From the data shown in Figure 3C, as catalyst loading increased, the conversion rate of sesamin increased, indicating that there was no mass-transfer limitation in this test. The reason might be that the more active sites provided by the catalyst, the faster the conversion rate (35). At the reaction time of 240 min, the sesamin conversion was 16.38% at 1.0% catalyst loading, 35.78% at 1.6% catalyst loading, and 54.78% at 3.0% catalyst loading.

### Effect of initial concentration of reactant

Figure 3D shows the effect of initial reactant concentration on the sesamin conversion under the following conditions: reaction temperature, 80°C; rotation speed, 300 rpm; catalyst loading, 1.6%. The results showed that the kinetic rate decreased slightly with the increase of the initial concentration of reactant. At the reaction time of 240 min, the sesamin conversion reached 35.78% at the reactant concentration of 21.47 mmol/L. The sesamin conversion was 33.34 and 32.27% at the reactant concentrations of 22.91 and 23.38 mmol/L, respectively. There was no significant difference among different initial concentrations of reactant. Sesamin was found to have poor solubility when it was added back to sesame oil (26). In this test, it was assumed that sesamin was dissolved in the sesame oil when no crystallization of sesamin in the sesame oil was observed with the naked eye. As for the phenomenon that the conversion rate was relatively lower when the concentration of reactant was slightly higher, it is possible that there were crystals blocking the active sites on the catalyst surface (35).

### Variation of peak areas in liquid chromatograms

In order to monitor the evolution of sesamin and asarinin, samples withdrawn at different times during the reaction were analyzed by HPLC-UV. The peak at 17 min corresponds to the substance sesamin, and the peak at 19 min is asarinin. It was observed intuitively from Figure 3E that the peak area of sesamin was decreased gradually and asarinin increased with the reaction time going on. This was interpreted to mean that, as the reaction proceeded, sesamin was partially converted to asarinin. These results are consistent with previous studies (8, 9).

### Reusability and characterization of citric acid loaded on Hβ

**FIGURE 4:**
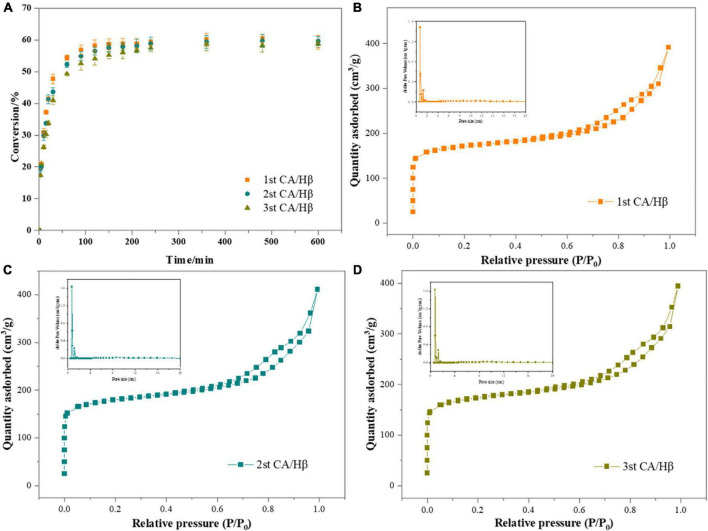
**(A)** The sesamin conversion with recycled catalyst. Reaction conditions: catalyst loading, 3.0%; sesame oil temperature, 80°C; rotation speed, 300 rpm; **(B–D)** N_2_ adsorption and desorption isotherms of recycled catalyst.

Easy recycle and reusability are the main advantage of heterogenous catalysts (36). The reusability of the CA/Hβ catalyst was evaluated by the sesamin conversion reaction at a temperature of 80°C and rotation speed of 300 rpm, with a catalyst loading of 3.0%. As shown in Figure 4A, the CA/Hβ catalyst exhibited good recyclability in the sesamin conversion, the sesamin conversion decreased insignificantly, from 59.21 to 57.49% at 240 min.

The textural characteristics of catalyst was assessed by N_2_ adsorption and desorption isotherm analysis (Figures 4B–D) (28, 37). According to the classification of international union of pure and applied chemistry (IUPAC), the isotherm of the catalysts displayed a combination of type I and type IV isotherms. It is clear that, at lower relative pressures (*P*/*P*_0_ = 0.0–0.1), the catalysts showed isotherm type I, which corresponds to microporous material. A hysteresis loop was observed at the upper section of isotherm over the relative pressure range from 0.7 to 1.0, indicating that there were mesopores in the solid. This is further demonstrated in the BET analysis results in Table 2.

**TABLE 2:** Surface properties of catalysts.

	BET surface area/m^2^/g	External surface area m^2^/g	Pore size (nm)	Pore volume (cm^3^/g)	Micropore volume (cm^3^/g)	Mesopore volume (cm^3^/g)
1st CA/Hβ	552.48	140.79	4.39	0.61	0.20	0.40
2st CA/Hβ	579.38	147.27	4.39	0.64	0.21	0.42
3st CA/Hβ	560.62	143.46	4.35	0.61	0.20	0.41

The structural properties including surface area, pore volume and pore size of reused catalysts were summarized in Table 2) (38). After the catalyst was calcined at 500°C for 4 h, the specific surface area, pore size and pore volume renew to that of the fresh catalyst, and the seamin conversion was almost the same as that of the fresh catalyst (Table 2 and Figure 4A). It shows that calcination could remove the most of the organic substance adsorbed on the catalyst. A slight descend of pore size was observed in each recycle of the catalyst, which was consistent with the reduction of sesamin conversion. It may be that the effect of separation and n-hexane washing on the catalyst, and part of the active site of the catalyst is blocked by organic substance (35). In brief, these results revealed that the catalytic activity of the regenerated catalyst was almost the same as that of the fresh catalyst used for the first time (1st). The catalytic of catalyst could be reversed easily by calcination.

### Kinetics

According to the published literature (8, 9, 22), the sesamin conversion is reversible under certain conditions. The overall reaction can be represented as follows:


(3) $$A\overset{\text{CATALYST}}{⇌}B$$

Based on the mechanism in Figure 1, the conversion reaction can be described in three steps. The first step is the opening of sesamin’s furan ring with catalyst, forming intermediate *I*_1._ The second step is the intermediate *I*_1_ turning into an intermediate of asarinin and catalyst; the third step is the desorption of the intermediate of asarinin and catalyst to release asarinin and catalyst.


(4) $$\text{A}\overset{k_{1}}{\underset{k_{-1}}{⇌}}{\text{I}}_{1}$$

(5) $${\text{I}}_{1}\overset{k_{2}}{\underset{k_{-2}}{⇌}}{\text{B-}}^{*}$$

(6) $${\text{B-}}^{*}\overset{k_{3}}{⇌}{\text{B+}}^{*}$$

where A is sesamin, B is asarinin, *K*_1_, *K*_2_, and *K*_3_ are the equilibrium constants; [*I_1_*] is the concentrations of intermediate *I*_2_, mol/L; [*] is the concentration of the catalytic active sites at time *t*, mol/L, [B−*] represents the product of asarinin coupling with the active sites of catalyst at time *t*, mol/L. Assuming that the second step is the rate-determining step, the kinetics of the conversion reaction can be expressed as:


(7) $$R_{\text{Iso}}=R_{2}=\left(k_{2}\,\left[I_{1}\right]-k_{-2}\,\left[\text{B}\right]\right)\,w_{\text{cat}}$$

When the quasi-equilibrium approximation is applied to the intermediates,


(8) $$K_{1}=\frac{\text{[}I_{1}]}{\left[\text{A}\right]\,\left[*\right]},K_{2}=\frac{\left[B-*\right]}{\text{[}I_{1}]}\,a\,n\,d\,K_{3}=\frac{\left[\text{B}\right]\,\left[*\right]}{\left[B-*\right]}$$

The concentration of intermediate *I*_*1*_ can be expressed as


(9) $$\left[I_{1}\right]=K_{1}\,\left[\text{A}\right]\,\left[*\right]$$

By combining Eqs 6, 7, the conversion kinetics become


(10) $$R_{\text{Iso}}=\left(k_{2}\,K_{1}\,\left[\text{A}\right]\,\left[*\right]-k_{-2}\,\left[\text{B}\right]\right)\,w_{\text{cat}}$$

where *w*_*Cat*_ is the catalyst loading in g/L.

### Mass balance

Experiments were carried out in a vacuum. We assumed *K*_1_ = *K*_2_, and that the mass balance applied to the surface species leads to


(11) $${\left[*\right]}_{0}=\left[*\right]+\text{[}I_{1}]+[\text{B}-*]$$

where, [*]_0_ is the total concentration of the catalytic active sites, mol/L. The concentration of active sites at time *t* is


(12) $$\left[*\right]=\frac{{\left[*\right]}_{0}}{1+K_{1}\,\left[\text{A}\right]+K_{2}\,\left[\text{B}\right]}$$

When the following notations are introduced: *k*_*Iso*_ = *K*_1_*k*_2_, *K*_*Iso*_ = *k*_2_/*k*-_2_, Eq. 6 becomes


(13) $$R_{\text{Iso}}=k_{\text{Iso}}\times \frac{w_{\text{cat}}}{1+K_{1}\,\left[A\right]+K_{2}\,\left[B\right]}\times \left(\left[A\right]-\frac{1}{K_{\text{Iso}}}\,\left[\text{B}\right]\right)$$

All the associated parameters were estimated during the kinetic modeling stage (39, 40). The following ODEs describe the material balances of the different components in this reaction system


(14) $$\frac{\text{d}\,\left[A\right]}{\text{d}\,t}=-R_{\text{Iso}}$$

(15) $$\frac{\text{d}\,\left[\text{B}\right]}{\text{d}\,t}=R_{\text{Iso}}$$

### Modeling

**FIGURE 5:**
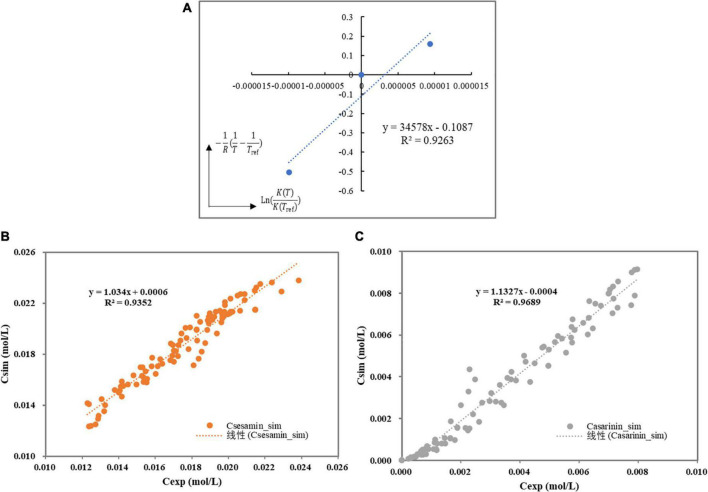
**(A)** Van’t Hoff curve for Henry’s constants. **(B)** Parity plot of experimental versus simulation of the sesamin concentration. **(C)** Parity plot of experimental versus simulation of the asarinin concentration.

To avoid correlation between the pre-exponential factor and activation energy, a modified Arrhenius equation (19, 21) was used to express the rate constants:


(16) $$k_{\text{Iso}}\,\left(T\right)=e\,x\,p\,\left[l\,n\,\left(k_{\text{Iso},\text{ref}}\right)+\frac{E_{\text{a}}}{R\cdot T_{\text{ref}}}\cdot \left(1-\frac{T_{\text{ref}}}{T}\right)\right]$$

where, *k*_*Iso*_ is the rate constant and *E_a_* is activation energy (kJ/mol); the gas constant 8.314 J/(mol/K) and reaction temperature (K) are represented by *R* and *T*, respectively. In total, 353.15 K was selected as the reference temperature (*T*_*ref*_). The equilibrium constant *K*_Iso_(*T*) can be expressed by Van’t Hoof law (41, 42).


(17) $$K_{\text{Iso}}\,\left(T\right)=e\,x\,p\,\left[l\,n\,K\,\left(T_{\text{ref}}\right)+\frac{△\,H_{r\,\mathrm{Iso}}}{R\cdot T_{\text{ref}}}\cdot \left(1-\frac{T_{\text{ref}}}{T}\right)\right]$$

where, *T*_*ref*_ represents the reference temperature (K) and △*H*_*r*Iso_ is the enthalpy for the sesamin conversion in kJ/mol. The kinetic constants (*ln*(*k*_*Iso*,*ref*_)) and $\frac{E_{\text{a}}}{R\,T_{\text{ref}}}$ were estimated. Thereafter, by plotting $l\,n\,\left(\frac{K\,\left(T\right)}{K\,\left(T_{\text{ref}}\right)}\right)$ versus $\left(\frac{1}{R}\,\left(\frac{1}{T}-\frac{1}{T_{\text{ref}}}\right)\right)$, it was demonstrated that this hypothesis is correct within the experimental temperature range. Figure 5A shows that the sesamin conversion in the sesame oil solution was endothermic (△*H*_*r*Iso_ = 34.578*kJ*/*mol*).

The estimated kinetic parameters for sesamin conversion and their HPD intervals are shown in Table 3. The HPD intervals for the estimated values are less than 20%, indicating that the parameter estimation for this model was accurate.

**TABLE 3:** Estimated and statistical data at *T*_ref_ = 353.15*K* for sesamin conversion.

	Estimated value	95% marginal HPD intervals	HPD in %
Ln*k*_*Iso*_(*T*_ref_) (L mol^–1^ s^–1^)	9.74E+00	6.97E−02	0.72
*Ea*/(*RT*_*ref*_)	2.14E+01	3.91E+00	18.23
*K_1_*	4029.99	Indeterminate	–
*K*_*Iso*_ (T_*ref*_)	0.536	–	–
*H*_*Iso*_ (J mol^–1^)	34578	–	–

The correlation matrix for the kinetic parameters is represented in Table 4. It was observed that the correlations among different kinetic parameters are very low, indicating good reliability of the developed model.

**TABLE 4:** Correlation matrix of kinetics modeling.

	Lnk_I*so*_(T_*ref*_)	*E*a/*R*(T_*ref*_)	K_1_
Ln*k*_*Iso*_(*T*_ref_)	1		
*E*a/*RT*_*ref*_	−0.157	1	
*K_1_*	0	0	0

**FIGURE 6:**
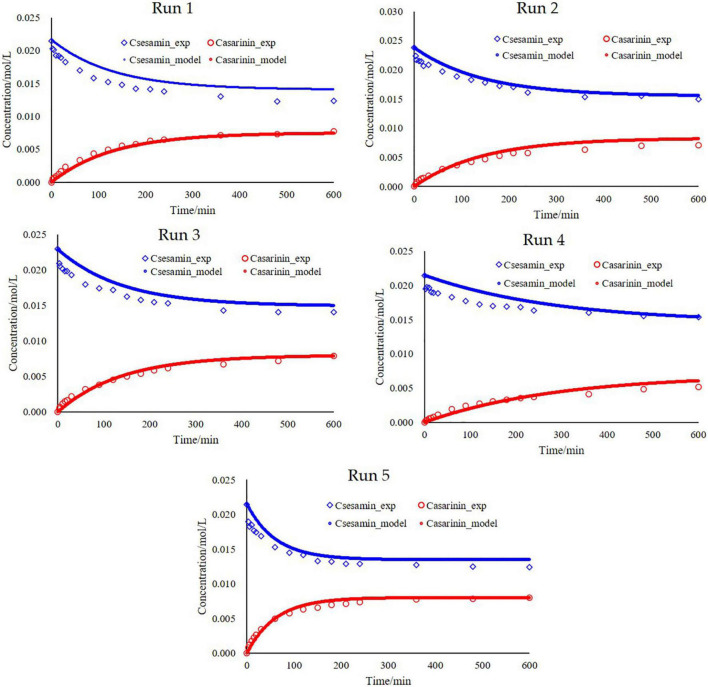
Fitting the model to the experiments and simulation of C_*sesamin*_ and C_*asarinin*_ for experiments 1–5. Run 1 [(C_*A*_) = 21.47 mmol/L with catalyst loading, 1.6%; temperature, 80°C; rotation speed, 300 rpm and (C_*B*_) = 0 mmol/L]; Run 2 [(C_*A*_) = 23.83 mmol/L with catalyst loading, 1.6%; temperature, 80°C; rotation speed, 300 rpm and (C_*B*_) = 0 mmol/L]; Run 3 [(C_*A*_) = 22.91 mmol/L with catalyst loading, 1.6%; temperature, 80°C; rotation speed, 300 rpm and (C_*B*_) = 0 mmol/L]; Run 4 [(C_*A*_) = 21.47 mmol/L with catalyst loading, 1.6%; temperature, 70°C; rotation speed, 300 rpm and (C_*B*_) = 0 mmol/L]; Run 5 [(C_*A*_) = 21.47 mmol/L with catalyst loading, 1.6%; temperature, 90°C; rotation speed, 300 rpm and (C_*B*_) = 0 mmol/L].

By plotting the experimental concentrations versus the simulated values, one sees that the simulated values are close to the experimental ones with high linear correlation coefficients ($R_{S\,e\,s\,a\,m\,i\,n}^{2}$ = 0.93 and $R_{A\,s\,a\,r\,i\,n\,i\,n}^{2}$ = 0.97), indicating that it is in good agreement with experimental data and calculated values (Figures 5B,C). Fits of the model to the experimental observations are presented in Figure 6 (Run 1–5). The kinetic model fits the experimental data well over the entire reaction, and the explanation coefficient exceeds 90%. Nevertheless, some deviations were observed (Run 1, 3, and 4). This deviation could be explained by the failure of the model to accurately take into account temperature-dependent density of the oil reaction medium and the formation of intermediate products.

## Conclusion

In this study, the effects of rotation speed, reaction temperature, catalyst loading and reactant concentration on the kinetics of sesamin conversion have been investigated. The conversion of sesamin into asarinin in CSO was efficiently achieved using CA/Hβ as a catalyst. The conversion kinetics were enhanced significantly by increasing catalyst loading and reaction temperature, while the effect of rotation speed and initial concentration of reactant was found to be negligible. A kinetic model was developed to simulate the sesamin conversion process. This model was applied for a reaction temperature range of 70–90°C, catalyst loading range of 1.0–3.0%, initial reactant concentration range of 21.47–23.83 mmol/L. It was observed that the calculated data were in agreement with experimental values by parity plot ($R_{S\,e\,s\,a\,m\,i\,n}^{2}$ = 0.93 and $R_{A\,s\,a\,r\,i\,n\,i\,n}^{2}$ = 0.97), indicating the model fit the experimental data quite well. The sesamin conversion was found to be endothermic (△*H*_*r*Iso_ = 34.578*kJ*/*mol*). The CA/Hβ catalyst could be easily regenerated by calcination, and there was no obvious loss of catalytic activity when reused. In summary, this study lays the foundation for the scale-up of the production of asarinin from sesame oil using CA/Hβ as the catalyst.

## Data availability statement

The original contributions presented in this study are included in the article/supplementary material, further inquiries can be directed to the corresponding author.

## Author contributions

QY: investigation, data curation, and writing—original draft. X-SC: methodology, data curation, and project administration. SL: software and project administration. X-DW: funding acquisition and supervision. H-ML: supervision and project administration. C-XZ: investigation and resources. Y-XM: resources and funding acquisition. All authors contributed to the article and approved the submitted version.
